# Supplementary material for: Intercalated PtCo Electrocatalyst of Vanadium Metal Oxide Increases Charge Density to Facilitate Hydrogen Evolution
Source: Molecules. 2024 Mar 28;29(7):1518. doi: 10.3390/molecules29071518 (PMC11013459; doi:10.3390/molecules29071518)
Supplement: Supplementary file 1 [file molecules-29-01518-s001.zip › molecules-2869429-supplementary.pdf]

Supporting Information

Intercalated PtCo Electrocatalyst of Vanadium Metal Oxide Increases Charge Density to Facilitate Hydrogen Evolution

Jingjing Zhang [1], Wei Deng [1,], Yun Weng [2], Jingxian Jiang [1], Haifang Mao [1], Wenqian Zhang [1], Tiandong Lu [1], Dewu Long [3] and Fei Jiang [1,]**

[1] School of Chemical and Environmental Engineering, Shanghai Institute of Technology, Shanghai 201418, China

[2] State Key Laboratory for Modification of Chemical Fibers and Polymer Materials, College of Textile, Donghua University, Shanghai 201620, China

[3] Key Laboratory in Interfacial Physics and Technology, Shanghai Institute of Applied Physics, Chinese Academy of Sciences, Shanghai 201800, China

Characterization

SEM images were acquired through a Quanta 400 FEG scanning electron microscope executed at 20 kV. TEM (Transmission Electron Microscopy) and elemental mapping images were collected from a Talos F200X TEM at an acceleration voltage of 200 kV. X-ray powder diffraction (XRD) was presented utilizing a Rigaku Ultimate IV with Cu-Kα radiation (λ = 1.5418 Å), and a scanning rate of 5° min^{-1} from 10° to 80°. X-ray photoelectron spectra (XPS) were executed on a PHI 5000 Versaprobe XPS instrument by non-monochromatized Al-Kα X-ray as the excitation source. The EPR measurement was performed on Bruker EMXplus-6/1 at a modulation amplitude of 0.8 mT, a modulation frequency of 100 kHz, a conversion time of 50 ms. The test temperature was set at 70 K. At the experimental resolution of 0.15eV, UPS spectra were obtained by He discharge lamp (He I 21.22eV). To better observe the secondary electron cut-off from the UPS spectrum, the sample is biased at -10 V.

Electrochemical measurements

The polarization curves were collected by linear sweep voltammetry (LSV) in the range of -1.0 to 0.0V at a scan rate of 5 $mV·s^{-1}$, with a scan rate of 100 $mV·s^{-1}$ for 5000 cycles, and the electrochemical stability of the catalyst was determined by cyclic voltammetry (CV) for 5000 cycles. Electrochemical impedance spectroscopy (EIS) was performed in the frequency range from 500 kHz to 1Hz at an overpotential (η = 200 mV). The electrochemical properties of the materials were tested at room temperature using 1.0 M potassium hydroxide solution at an electrochemical

workstation (CHI 660E) and a three-electrode system. The working electrode is made by distributing the catalyst in a 5.0 mm diameter glass carbon (GC) electrode. Ag/AgCl electrode was the reference electrode, and platinum plate electrode was utilized as the opposite electrode. The measured potential can be converted to RHE according to the formula ($E_{RHE} = E_{Ag/AgCl} + 0.0592pH + 0.197$).

Catalyst Preparation

Preparation of catalyst: First, 4 mg of prepared electrocatalyst and 30 μL of 5 wt% nafion solution were dispersed in a mixed solution of 1 mL of ethanol and water (1:3), ultrasonica for 15 minutes to form a mixed ink. And then, 10 μL of the prepared ink mixture was dispersed on a 5 mm diameter glassy carbon electrode and dried naturally. Therefore, the loading capacity of the sample is 0.197 mg cm^{-2}.

DFT calculations

The density functional theory (DFT) calculations were performed using the software of Materials Studio (MS)[1]. The energy cutoff (400 eV) of the eigen-function plane wave expansion and the 2×2×1 Monkhorst-Pack meshes are used to ensure the enthalpy convergence satisfyingly. For HER, in order to correctly describe the binding configuration and energy of H on PtCoVO catalyst, its hydrogen adsorption energy (ΔE_H) can be calculated by the following formula:

$$\Delta E_{H^*} = E_{H^*} - E^* - 1/2E_{H2} \quad (1)$$

where, * denotes the catalyst, E_{H^*}, E^* and $1/2E_{H2}$ represent the total energy of the catalyst with adsorbed H atom, catalyst and H_2 gas, separately.

The Gibbs free energy (ΔG_H*) for the H absorption was identified as:

$$\Delta G_H^* = \Delta E_H^* + \Delta ZPE - T\Delta S \quad (2)$$

where ΔZPE and ΔS are zero point energy (ZPE) change and entropy change (T = 298.15 K) between the adsorbed state and the gas phase of H* adsorption, separately.

The Gibbs free energy can be calculated as follows:

$$G_0 = E_{DFT} + ZPE - TS_0 \quad (3)$$

where G_0 stands for the Gibbs free energy, E_{DFT} represent the total adsorption energy, ZPE on behalf of the vibration energy, the change in entropy is represented by TS_0[2].

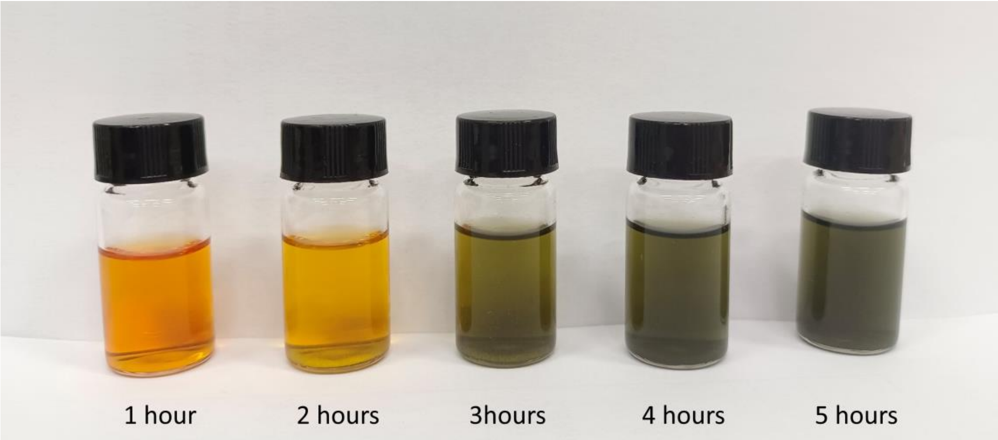

Figure S1. Digital image of vanadium powder in hydrogen peroxide ethanol solution at hourly intervals.

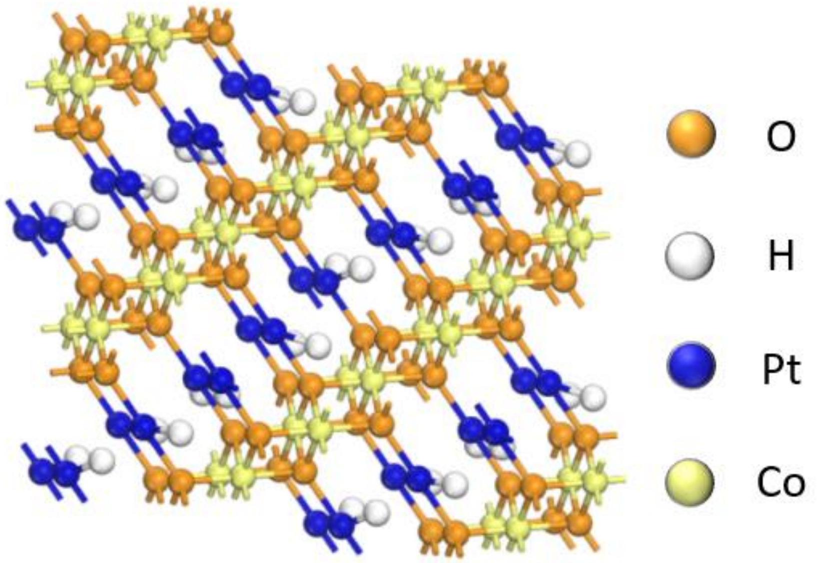

Figure S2. Structure diagram of PtCo(H*).

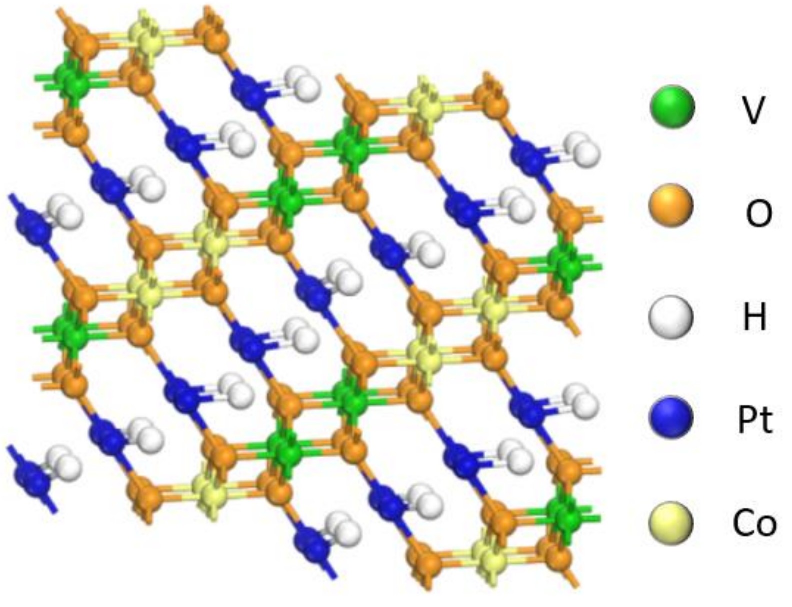

Figure S3. Structure diagram of PtCoVO(H*).

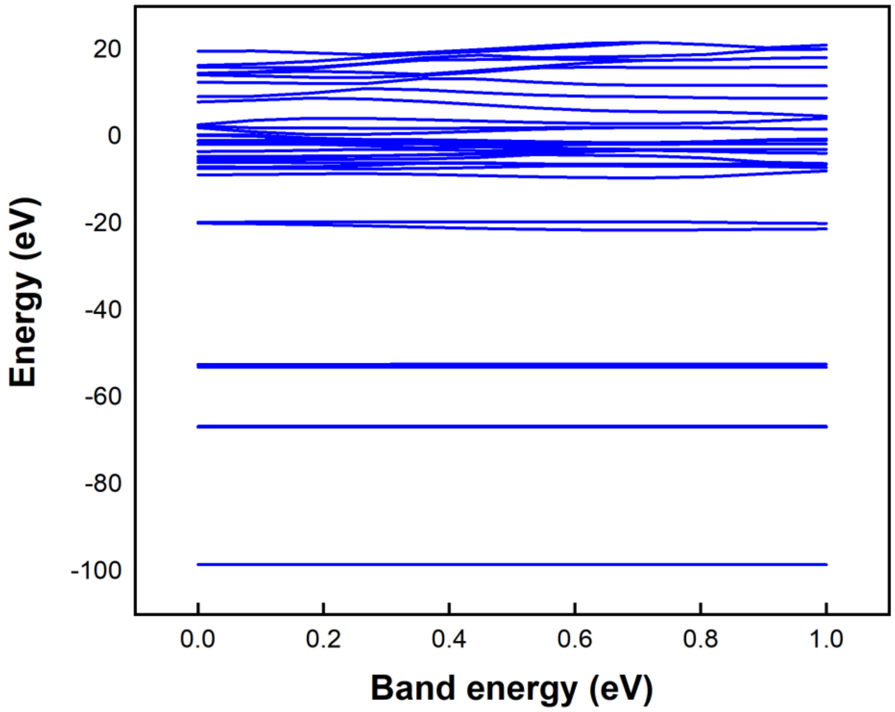

Figure S4. Calculated band structure of PtCo.

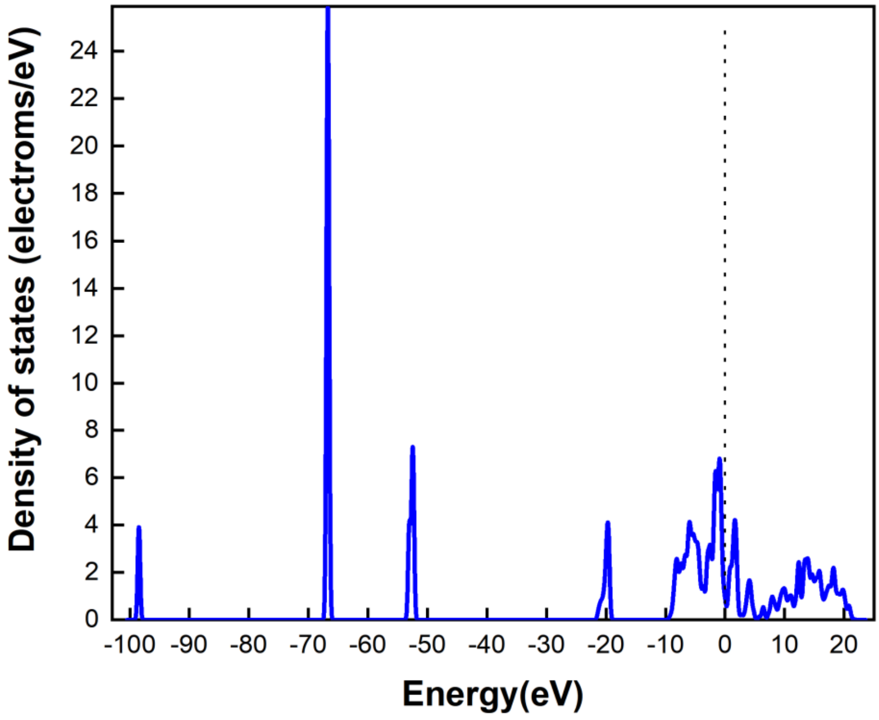

Figure S5. Calculated density of states (DOS) for PtCo.

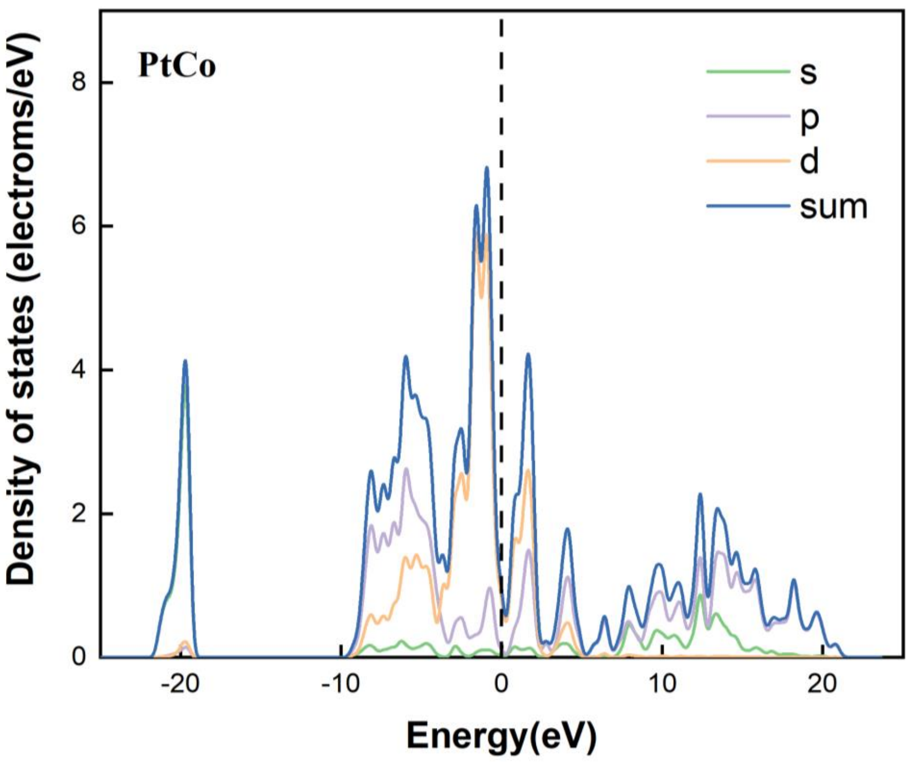

Figure S6. Calculated density of states (DOS) of PtCo.

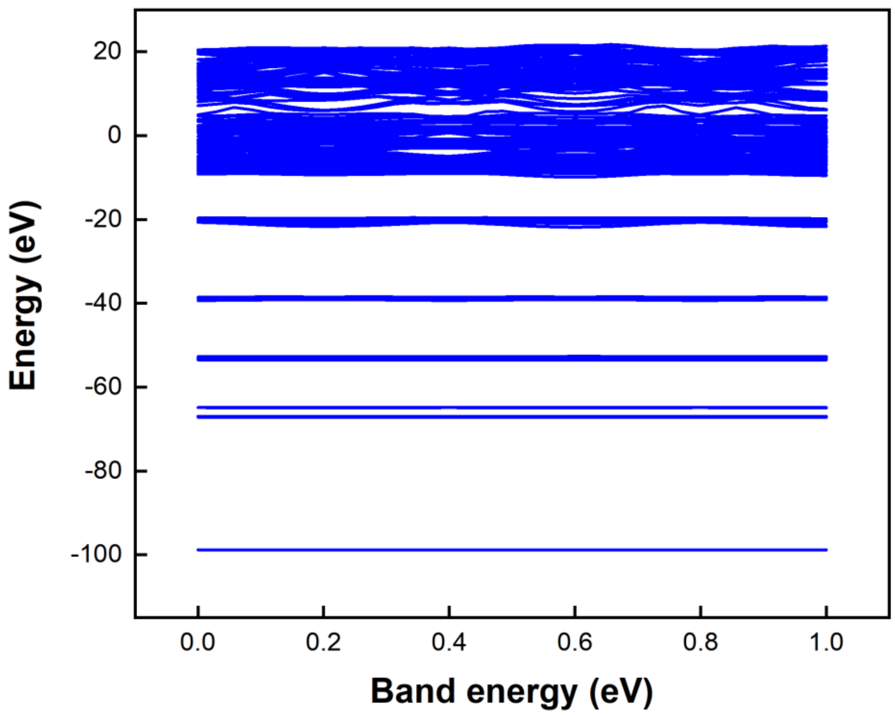

Figure S7. Calculated band structure of PtCoVO.

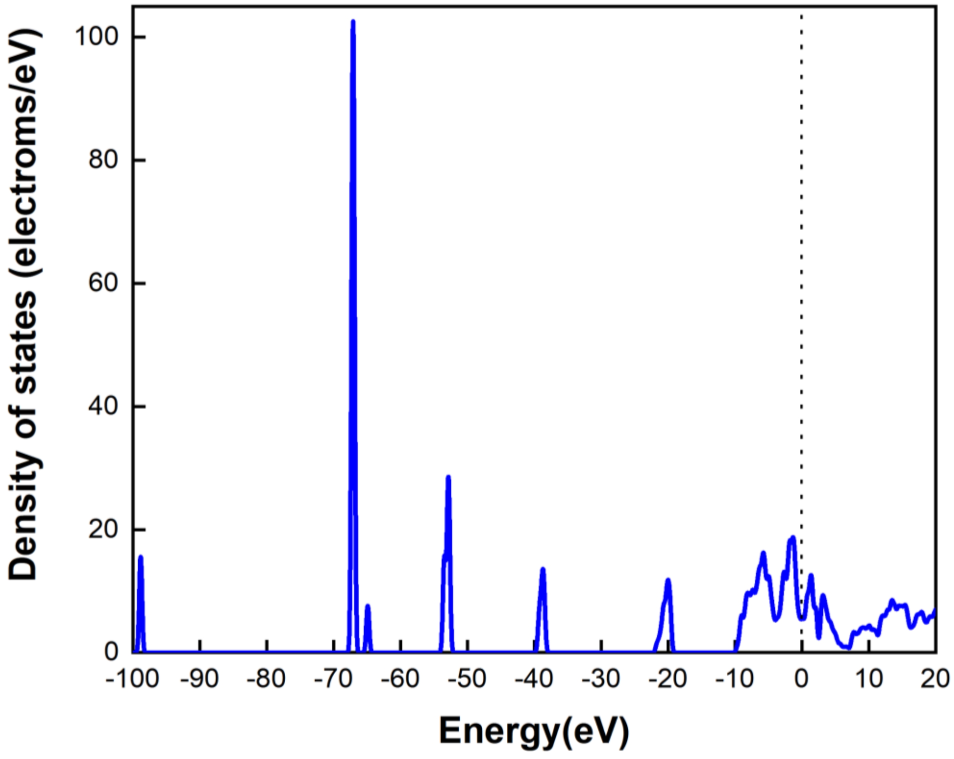

Figure S8. Calculated density of states (DOS) for PtCoVO.

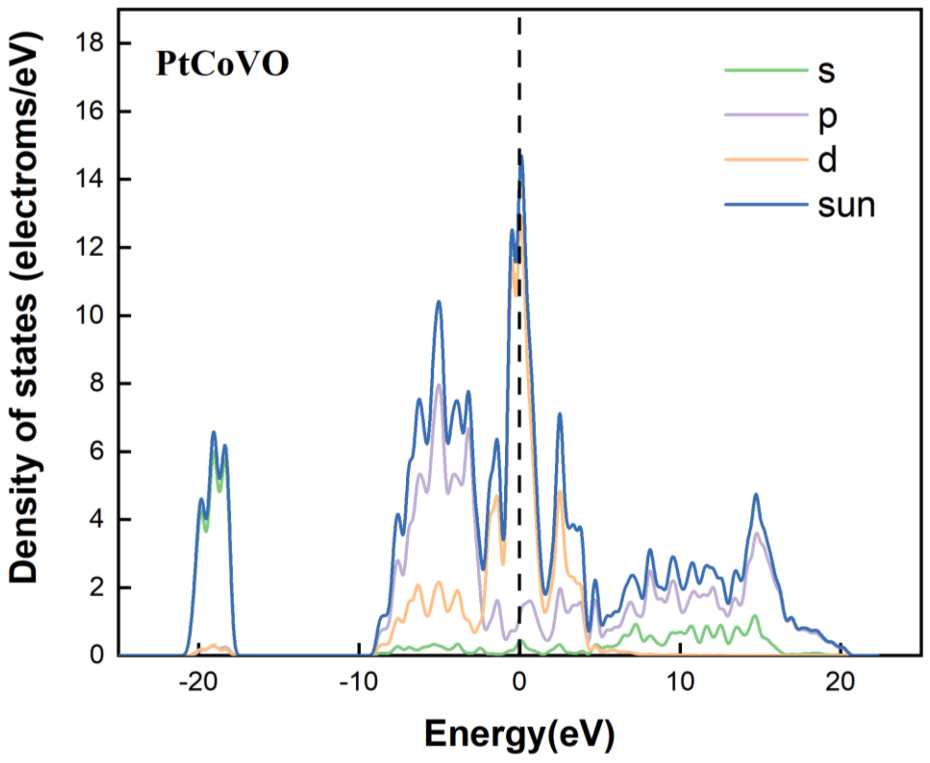

Figure S9. Calculated density of states (DOS) of PtCoVO.

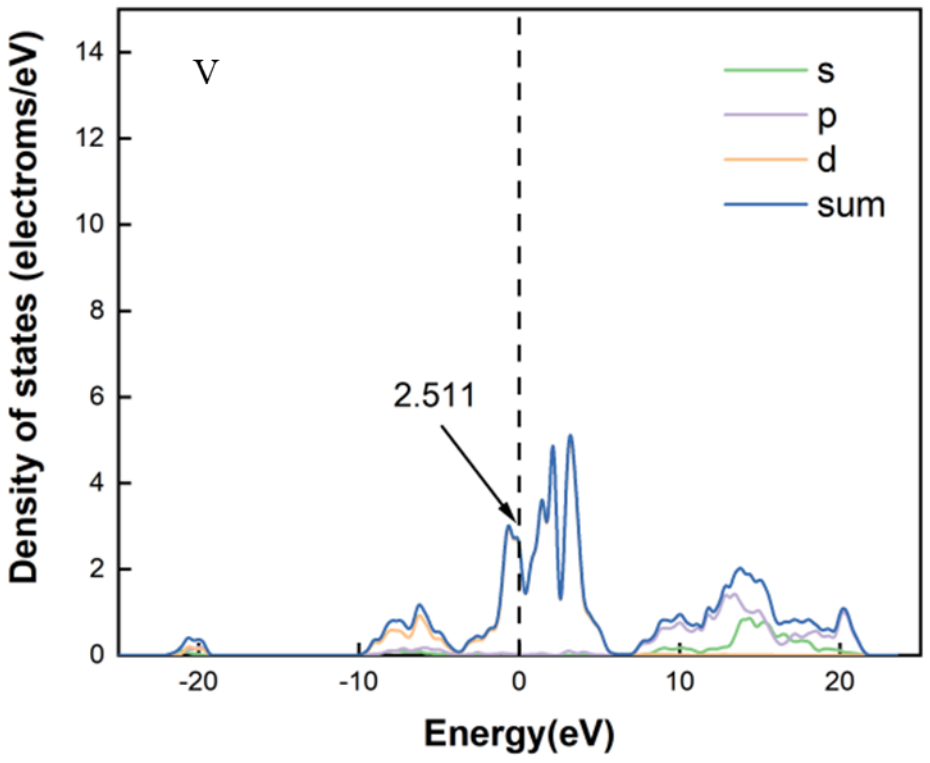

Figure S10. Calculated density of states (DOS) of V for PtCoVO.

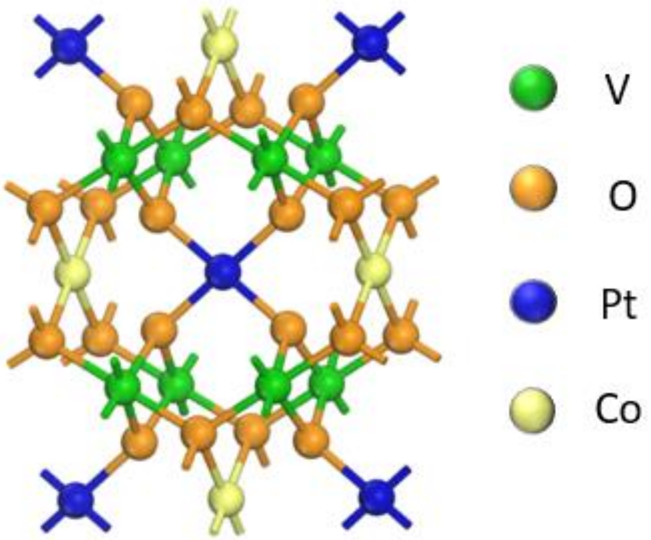

Figure S11. Structure diagram of PtCoV.

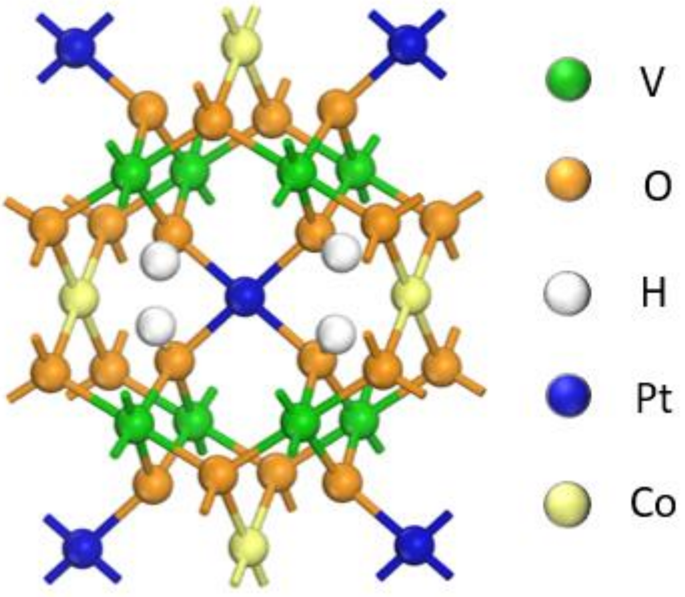

Figure S12. Structure diagram of PtCoV(H*).

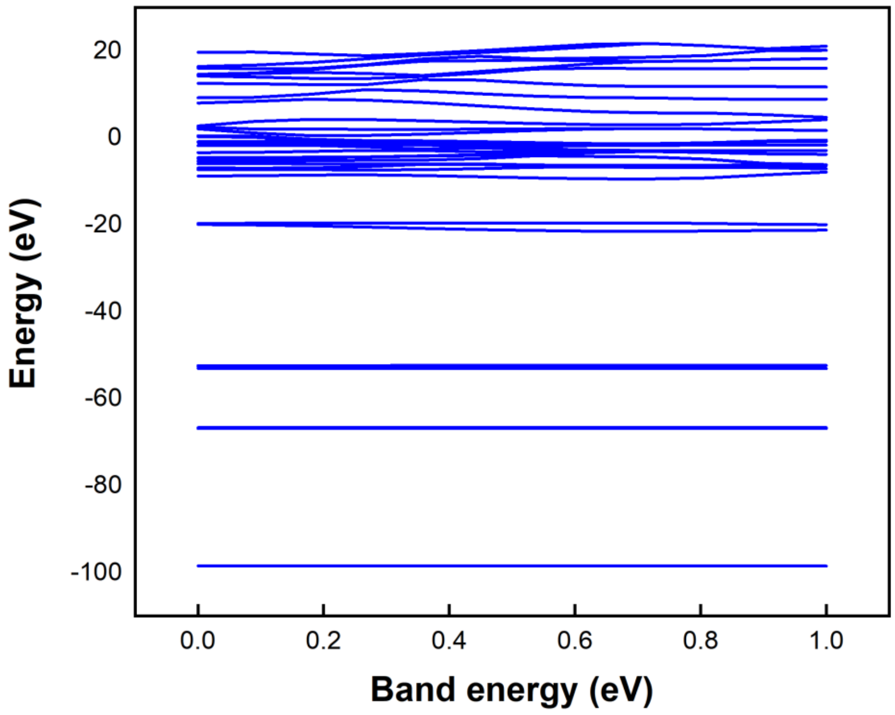

Figure S13. Calculated band structure of PtCoV.

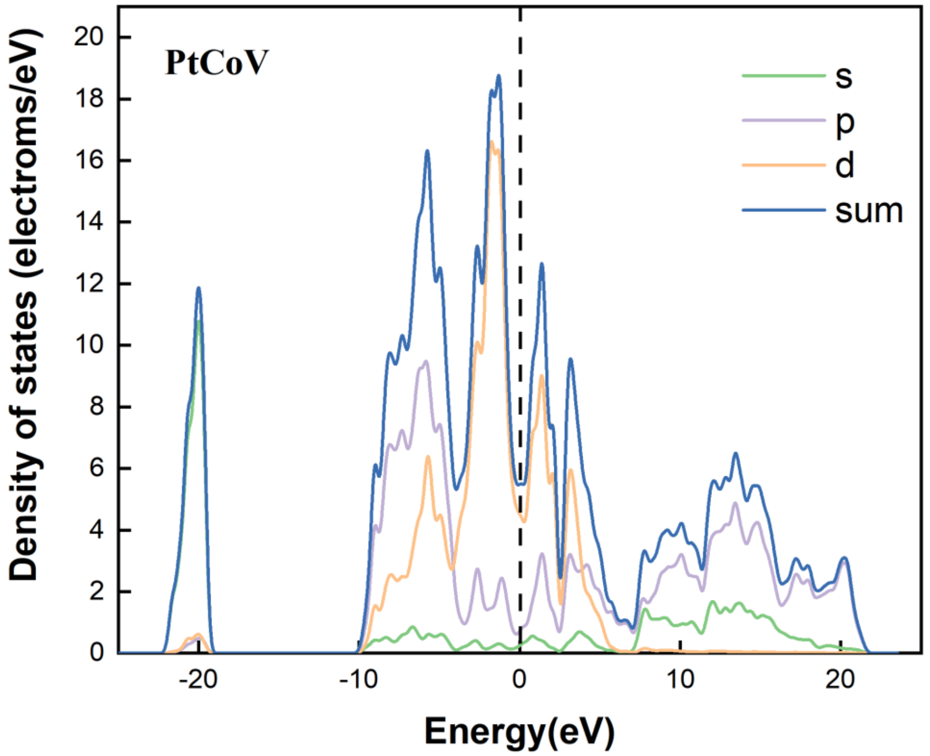

Figure S14. Calculated density of states (DOS) of V for PtCoV.

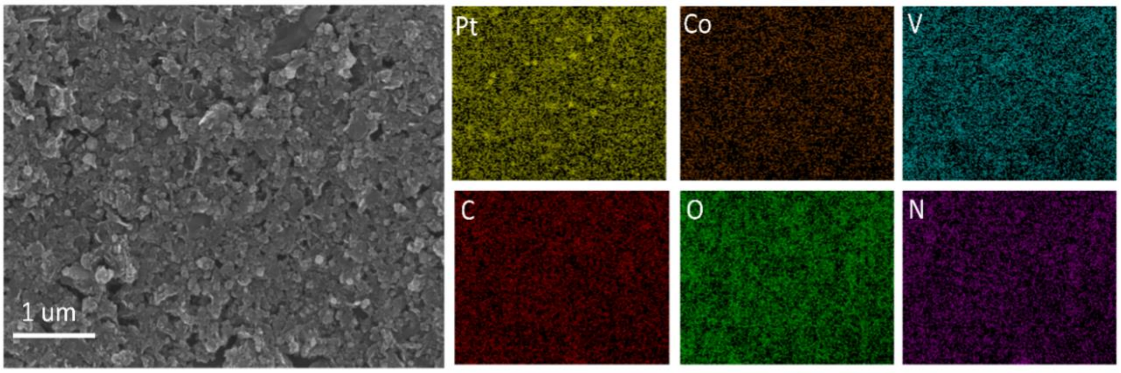

Figure S15. SEM images and corresponding mapping of PtCoVO/g-C_3N_4.

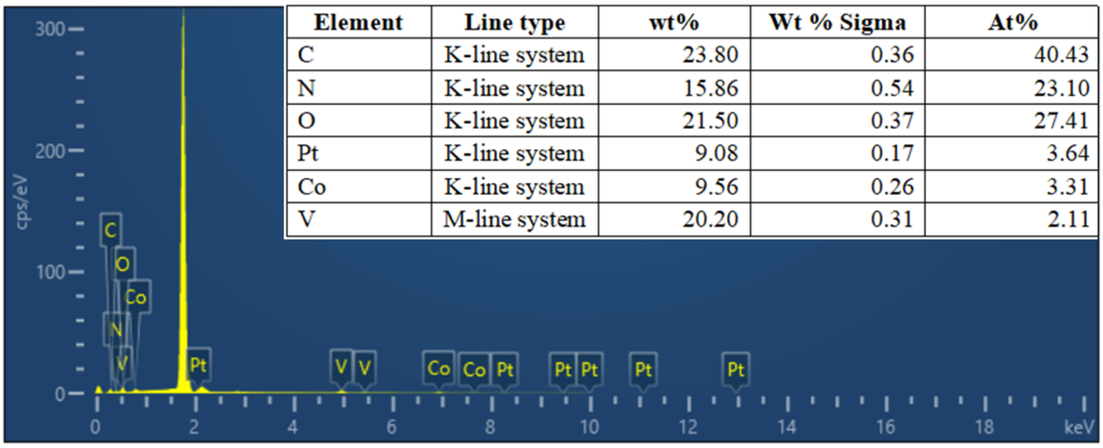

Element	Line type	wt%	Wt % Sigma	At%
C	K-line system	23.80	0.36	40.43
N	K-line system	15.86	0.54	23.10
O	K-line system	21.50	0.37	27.41
Pt	K-line system	9.08	0.17	3.64
Co	K-line system	9.56	0.26	3.31
V	M-line system	20.20	0.31	2.11

Figure S16. The distribution of the total spectrum of PtCoVO/g-C_3N_4.

Table S1. Inductively coupled plasma emission spectrometry (ICP-AES) was used to determine At % of elements in PtCoVO/g-C_3N_4.

Element	At%
**Pt**	**6.43**
**Co**	**7.02**
**V**	**15.95**

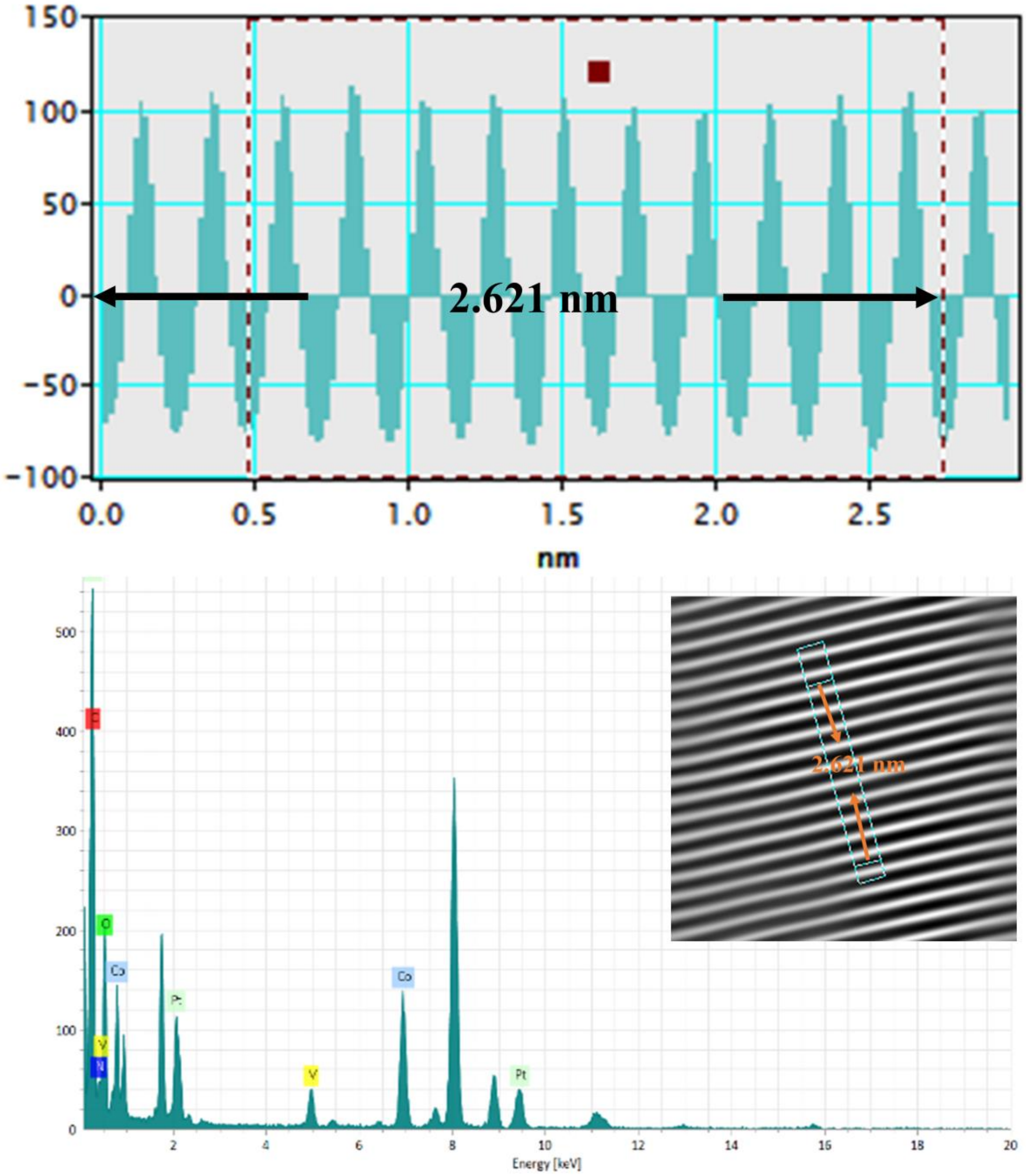

Figure S17. Spacing, EDS spectrum and IFFT of SEM for PtCoV/g-C_3N_4.

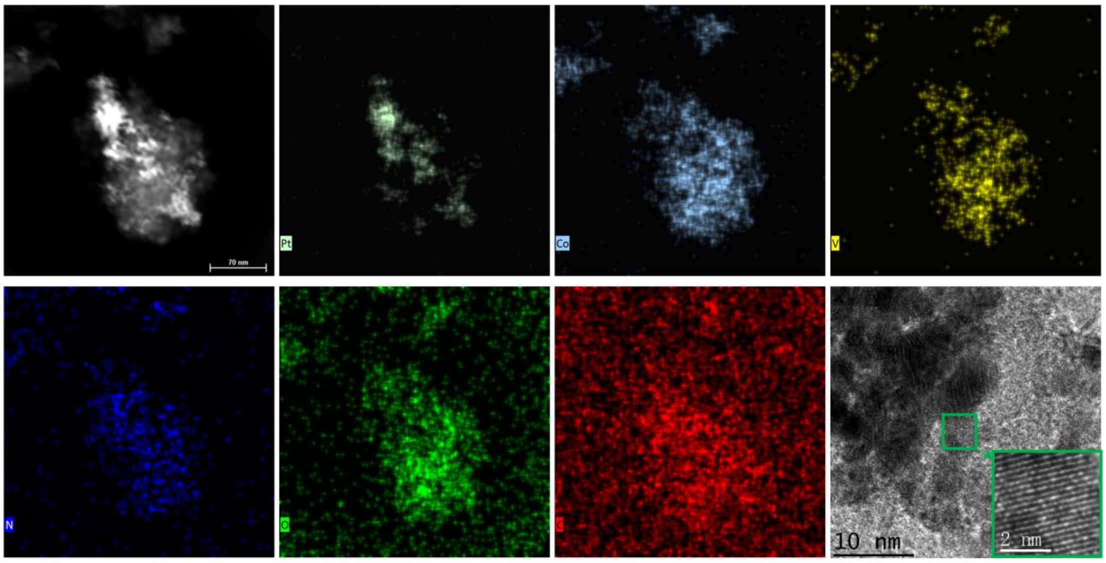

Figure S18. TEM image mapping of PtCoV/g-C_3N_4.

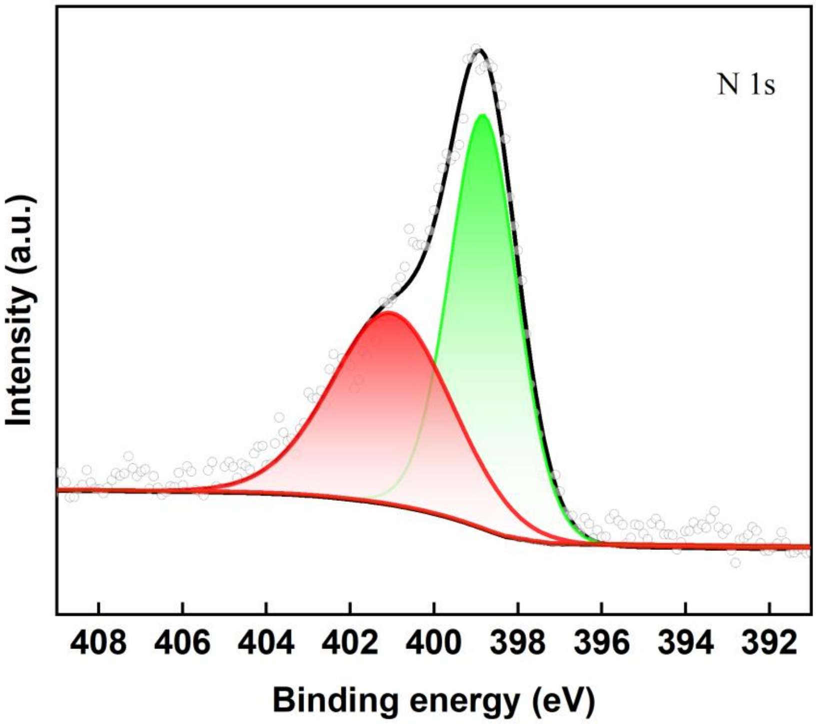

Figure S19. XPS spectra of N 1s for PtCoVO/g-C_3N_4.

Note S1. The work function was calculated according to the method reported by Liu[3], hν=21.22 eV.

$$\Phi = h\nu - (E_{fermi} - E_{cutoff})$$

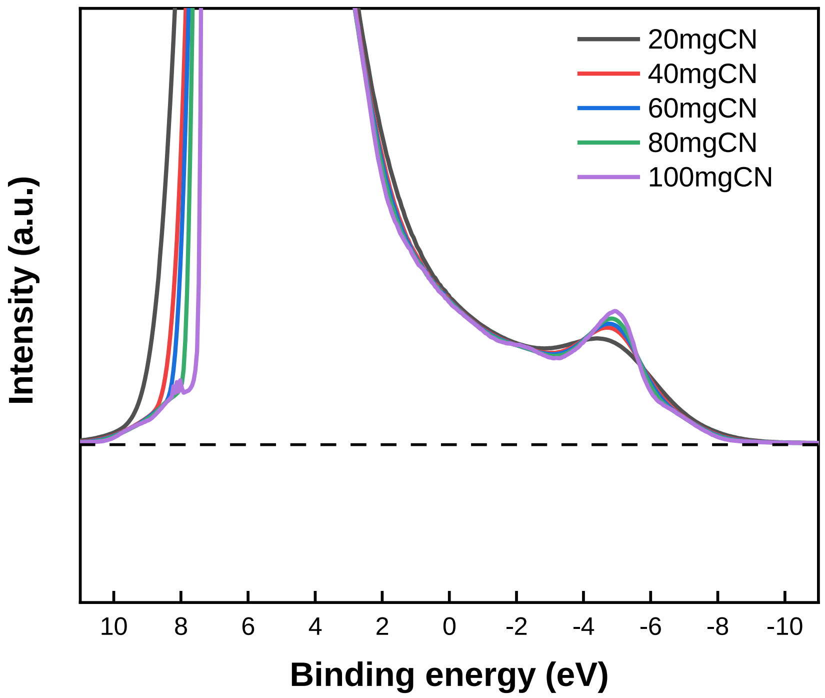

Figure S20. UPS spectra of PtCoVO/g-C_3N_4 under different amounts of g-C_3N_4 added.

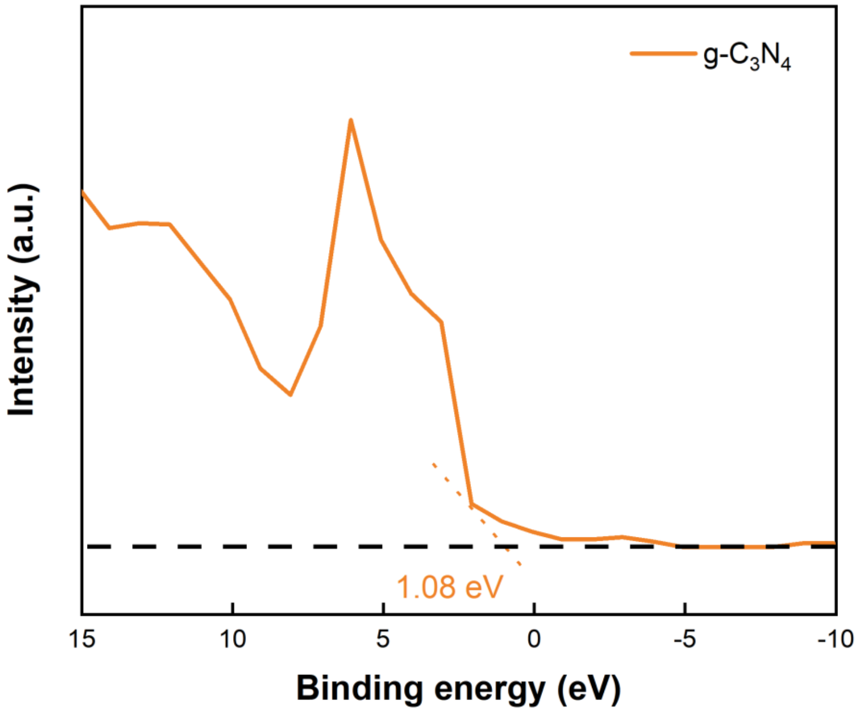

Figure S21. VB-XPS valence band spectra of g-C_3N_4.

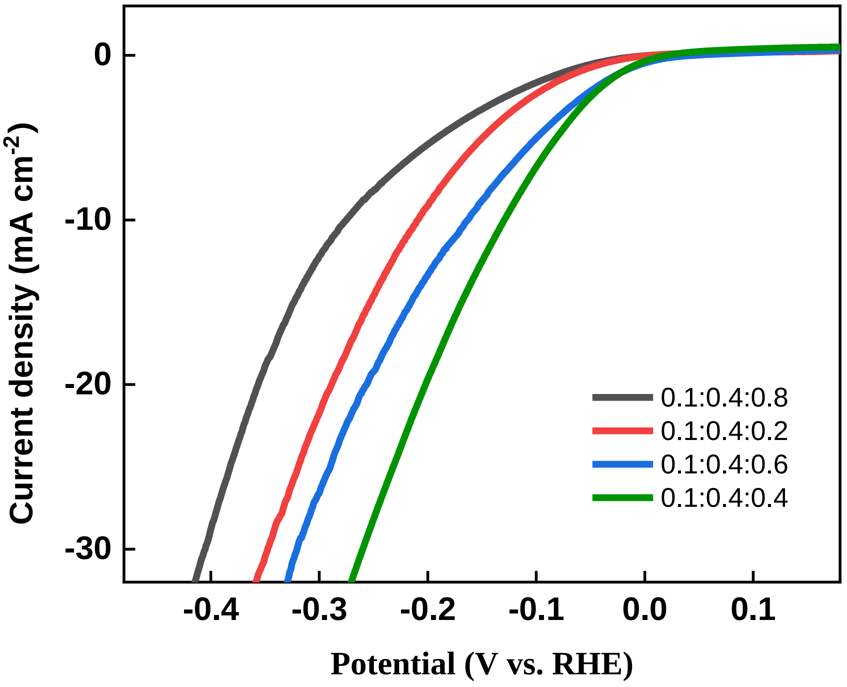

Figure S22. Polarization curves for different $H_xV_2O_5$ millimolar ratios of PtCoVO/g-C_3N_4.

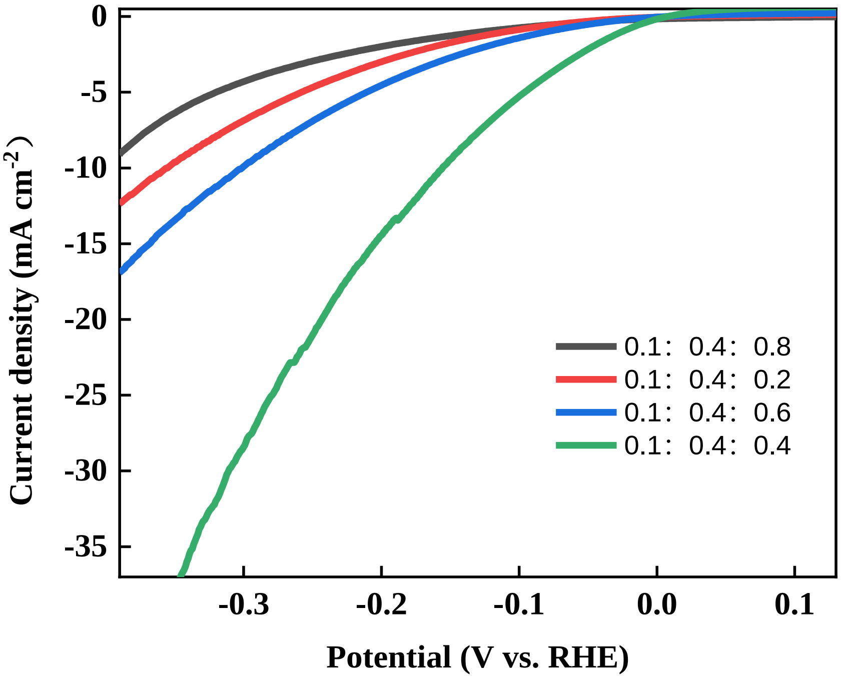

Figure S23. Polarization curves for different V powder millimolar ratios of PtCoVO/g-C_3N_4.

Table S2. Comparison of the reported electrocatalysts with our work of HER activity.

Catalyst	Electrolyte	Tafel slope (mV dec^{-1})	Overpotential at 10 mA cm^{-2} (mV)	Reference
PtCoVO/g-C_3N_4	1 M KOH	63.65	92	This work
$IrMo_{0.59}$ alloy	1 M KOH	50	23	[4]
Pt_2Ni_3-P	1 M KOH	66	44	[5]
Ru black	1 M NaOH	80	125	[6]
Co_2P@NPG	1 M KOH	96	165	[7]
$Mo_1N_1C_2$	0.1 M KOH	90	132	[8]
CoP/rGO-400	1 M KOH	38	150	[9]
CoOx@CN	1 M KOH	115	232	[10]
$FePO_4$/NF	1 M KOH	104.49	123	[11]
Mo_2N-Mo_2C/HGr	1 M KOH	68	154	[12]
MoB/g-C_3N_4	1 M KOH	46	133	[13]
MoC_x nano octahedrons	1 M KOH	59	151	[14]

Table S3. Comparison of the reported electrocatalysts with its hybrids of HER activity.

Catalyst	Electrolyte	Tafel slope (mV dec^{-1})	Overpotential at 10 mA cm^{-2} (mV)	Reference
$NiCo_2O_3@C_3N_4$	1 M KOH	146	89	[15]
$NiCo_2O_3$	1 M KOH	150	185	[15]
$WO_2@C_3N_4$	1 M KOH	94.4	98	[16]
WO_2	1 M KOH	173.32	526	[16]
$CuSCN/C_3N_4$	1 M KOH	47	85	[17]
CuSCN	1 M KOH	53	187	[17]

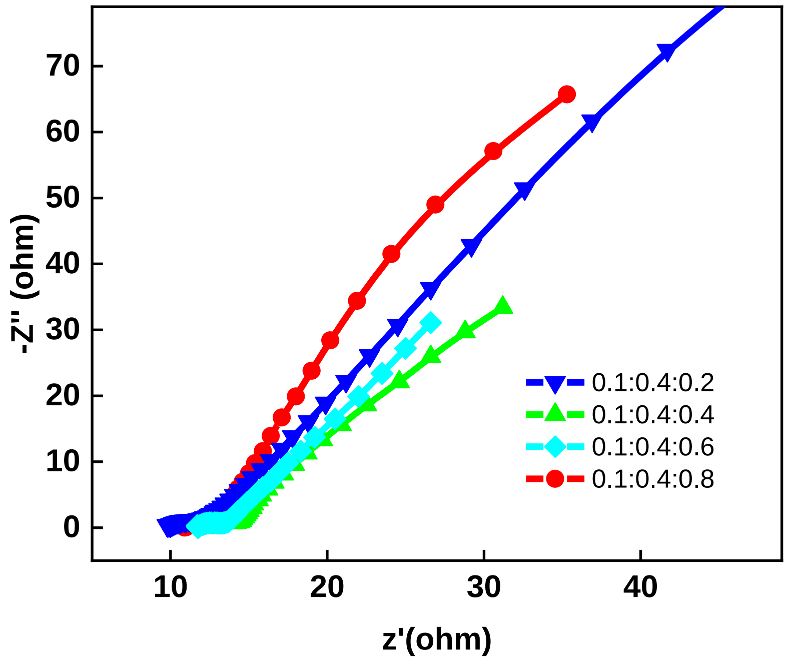

Figure S24. EIS Nyquist plots for different $H_xV_2O_5$ millimolar ratios of $PtCoVO/g-C_3N_4$.

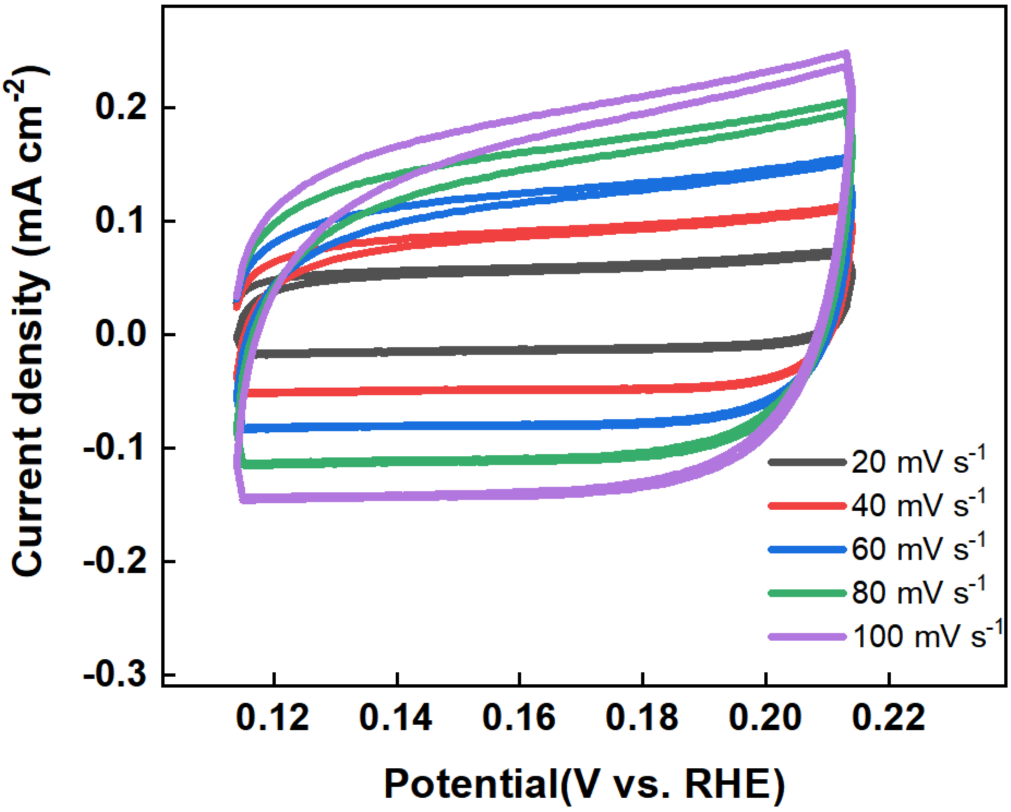

Figure S25. CV curves of PtCoVO/g-C_3N_4 in 1.0 M KOH solution at different scan rates.

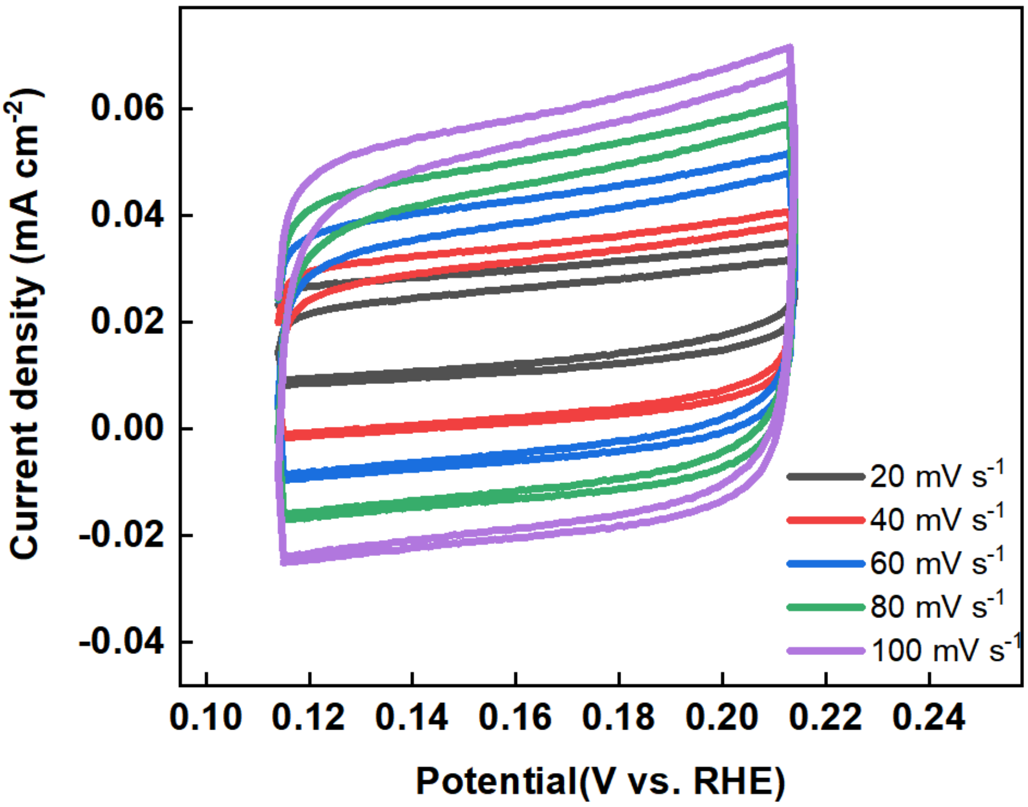

Figure S26. CV curves of PtCo/g-C_3N_4 in 1.0 M KOH solution at different scan rates.

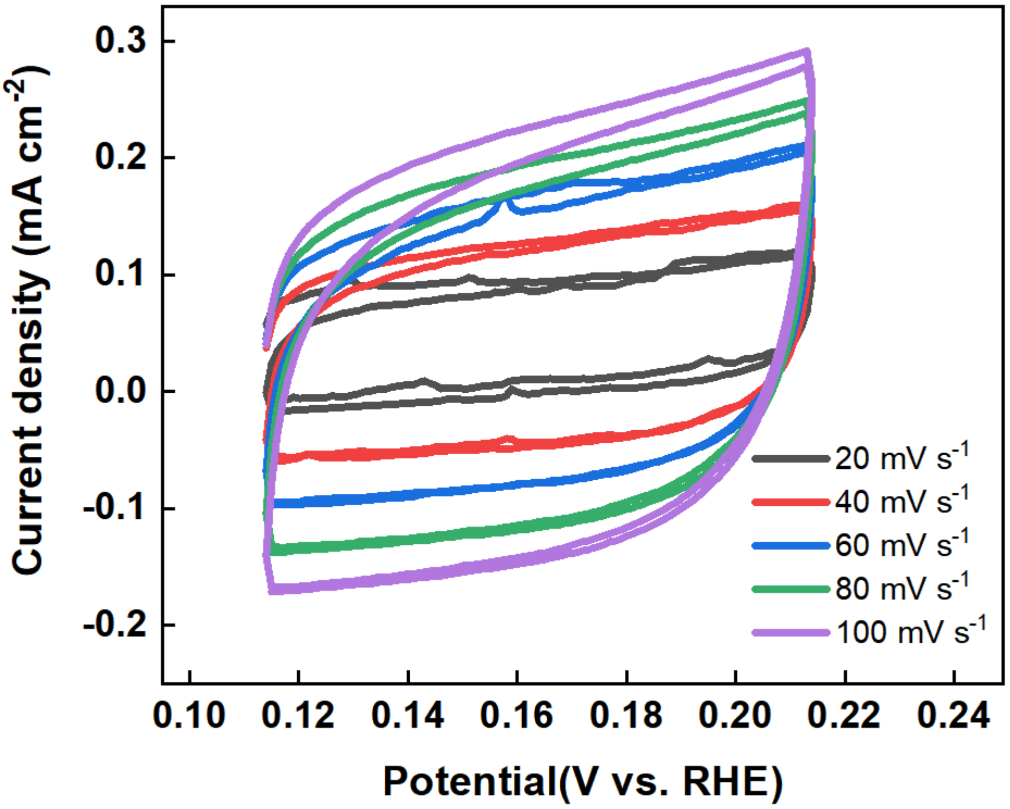

Figure S27. CV curves of PtCoV/g-C_3N_4 in 1.0 M KOH solution at different scan rates.

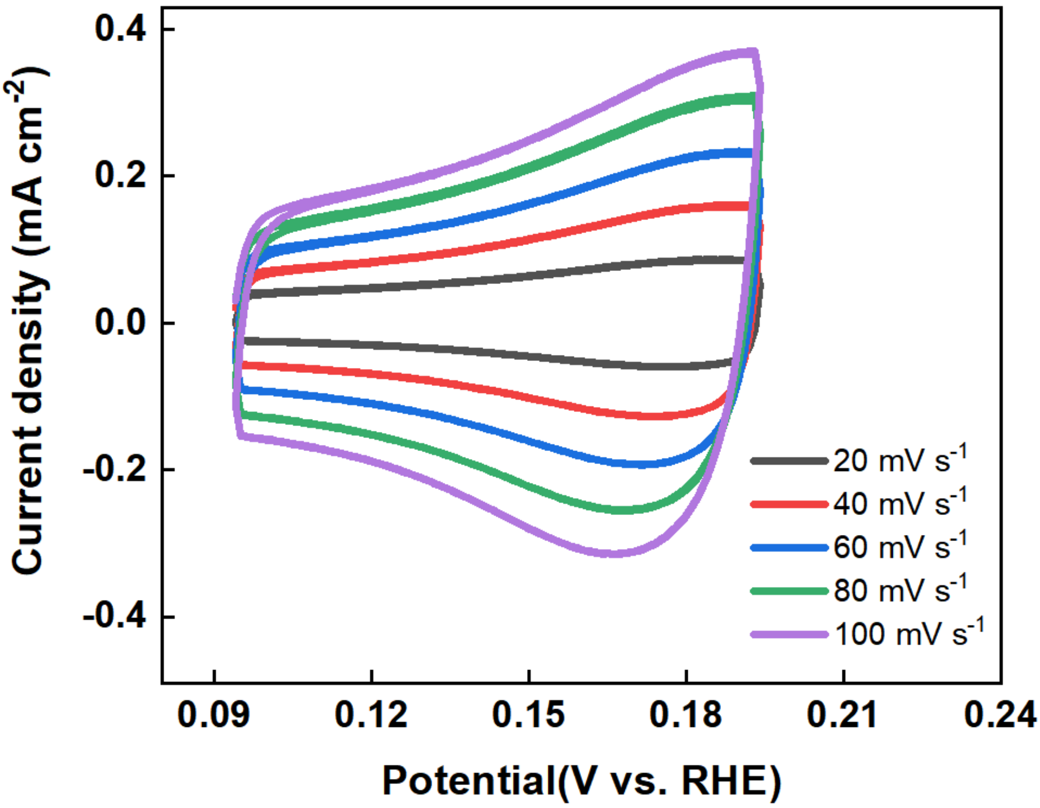

Figure S28. CV curves of Pt/C 20% in 1.0 M KOH solution at different scan rates.

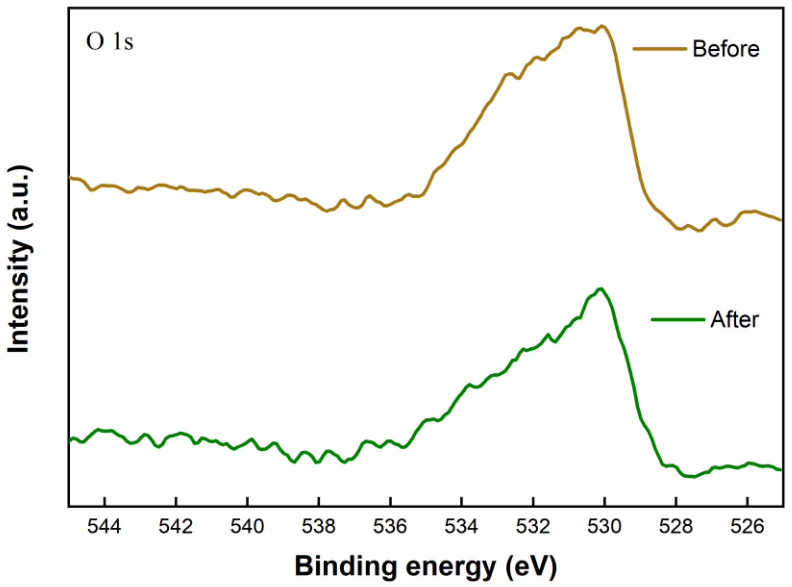

Figure S39. O 1s XPS spectra before and after electrochemical test of PtCoVO/g-C_3N_4.

References

[1] Rao, F., Ding, K., Zhou, Y., Zheng, Y., Xia, M., Lv, S., ... & Ma, E. Reducing the stochasticity of crystal nucleation to enable subnanosecond memory writing. *Science*, 2017. 358(6369), 1423-1427. https:// doi.org/10.1126/science.aao3212.

[2] Anand, R., Nissimagoudar, A. S., Umer, M., Ha, M., Zafari, M., Umer, S., ... & Kim, K. S. Late transition metal doped MXenes showing superb bifunctional electrocatalytic activities for water splitting via distinctive mechanistic pathways. *Advanced Energy Materials*, 2021. 11(48), 2102388. https://doi.org/10.1002/aenm.202102388.

[3] Liu, J., Liu, Y., Liu, N., Han, Y., Zhang, X., Huang, H., ... & Kang, Z. Metal-free efficient photocatalyst for stable visible water splitting via a two-electron pathway. *Science*, 2015. 347(6225), 970-974. https://doi.org/10.1126/science.aaa3145.

[4] Fu, L., Li, Y., Yao, N., Yang, F., Cheng, G., & Luo, W. IrMo nanocatalysts for efficient alkaline hydrogen electrocatalysis. *ACS Catalysis*, 2020. 10(13), 7322-7327. https://doi.org/10.1021/acscatal.0c02254.

[5] Wang, P., Shao, Q., Guo, J., Bu, L., & Huang, X. Promoting alkaline hydrogen evolution catalysis on P-decorated, Ni-segregated Pt–Ni–P nanowires via a synergetic cascade route. *Chemistry of Materials*, 2020. 32(7), 3144-3149. https://doi.org/10.1021/acs.chemmater.0c00172.

[6] Creus, J., Drouet, S., Surinach, S., Lecante, P., Collière, V., Poteau, R., ... & Sala, X. Ligand-capped Ru nanoparticles as efficient electrocatalyst for the hydrogen evolution reaction. *ACS Catalysis*, 2018. 8(12), 11094-11102. https://doi.org/10.1021/acscatal.8b03053.

[7] Zhuang, M., Ou, X., Dou, Y., Zhang, L., Zhang, Q., Wu, R., ... & Luo, Z. Polymer-embedded fabrication of Co_2P nanoparticles encapsulated in N, P-doped graphene for hydrogen generation. *Nano letters*, 2016. 16(7), 4691-4698. https://doi.org/10.1021/acs.nanolett.6b02203.

[8] Chen, W., Pei, J., He, C. T., Wan, J., Ren, H., Zhu, Y., ... & Li, Y. Rational design of single molybdenum atoms anchored on N-doped carbon for effective hydrogen evolution reaction. *Angewandte Chemie*, 2017. 129(50), 16302-16306. https://doi.org/10.1002/ange.201710599.

[9] Jiao, L., Zhou, Y. X., & Jiang, H. L. Metal–organic framework-based CoP/reduced graphene oxide: high-performance bifunctional electrocatalyst for overall water splitting. *Chemical Science*, 2016. 7(3), 1690-1695. https://doi.org/10.1039/C5SC04425A.

[10] Jin, H., Wang, J., Su, D., Wei, Z., Pang, Z., & Wang, Y. In situ cobalt–cobalt oxide/N-doped carbon hybrids as superior bifunctional electrocatalysts for hydrogen and oxygen evolution. *Journal of the American Chemical Society*, 2015. 137(7), 2688-2694. https://doi.org/10.1021/ja5127165.

[11] Yang, L., Guo, Z., Huang, J., Xi, Y., Gao, R., Su, G., ... & Dong, B. Vertical growth of 2D amorphous $FePO_4$ nanosheet on Ni foam: outer and inner structural design for superior water splitting. *Advanced Materials*, 2017. 29(46), 1704574. https://doi.org/10.1002/adma.201704574.

[12] Yan, H., Xie, Y., Jiao, Y., Wu, A., Tian, C., Zhang, X., ... & Fu, H. Holey reduced graphene oxide coupled with an Mo_2N–Mo_2C heterojunction for efficient hydrogen evolution. *Advanced Materials*, 2018. 30(2), 1704156. https://doi.org/10.1002/adma.201704156.

[13] Zhuang, Z., Li, Y., Li, Z., Lv, F., Lang, Z., Zhao, K., ... & Mai, L. MoB/g-C3N4 interface materials as a schottky catalyst to boost hydrogen evolution. *Angewandte Chemie*, 2018. 130(2), 505-509. https://doi.org/10.1002/ange.201708748.

[14] Wu, H. B., Xia, B. Y., Yu, L., Yu, X. Y., & Lou, X. W. Porous molybdenum carbide nano-octahedrons synthesized via confined carburization in metal-organic frameworks for efficient hydrogen production. *Nature communications*, 2015. 6(1), 6512. https://doi.org/10.1038/ncomms7512.

[15] Zhang, C., Wang, J., Liu, Y., Li, W., Wang, Y., Qin, G., & Lv, Z. Electrocatalytic HER Enhancement of C_3N_4 in $NiCo_2O_4$. *Chemistry–An Asian Journal*, 2022. 17(14), e202200377. https://doi.org/10.1002/asia.202200377.

[16] Lu, S. S., Zhang, L. M., Fan, K., Xie, J. Y., Shang, X., Zhang, J. Q., ... & Dong, B. In situ formation of ultrathin C_3N_4 layers on metallic WO_2 nanorods for efficient hydrogen evolution. *Applied Surface Science*, 2019. 487, 945-950. https://doi.org/10.1016/j.apsusc.2019.05.189.

[17] Zhao, Z., Yang, H., Zhu, Y., Luo, S., & Ma, J. Interfacial N–Cu–S coordination mode of CuSCN/C_3N_4 with enhanced electrocatalytic activity for hydrogen evolution. Nanoscale, 2019. 11(27), 12938-12945. https://doi.org/10.1039/C9NR02860A.
